# Effects of Kinesio Taping versus McConnell Taping for Patellofemoral Pain Syndrome: A Systematic Review and Meta-Analysis

**DOI:** 10.1155/2015/471208

**Published:** 2015-06-21

**Authors:** Wen-Dien Chang, Fu-Chen Chen, Chia-Lun Lee, Hung-Yu Lin, Ping-Tung Lai

**Affiliations:** ^1^Department of Sports Medicine, China Medical University, No. 91, Hsueh-Shih Road, Taichung City 404402, Taiwan; ^2^Department of Recreational Sport and Health Promotion, National Pingtung University of Science and Technology, No. 1, Shuefu Road, Neipu, Pingtung 91201, Taiwan; ^3^Division of Physical and Health Education, Center for General Education, National Sun Yat-sen University, No. 70 Lienhai Road, Kaohsiung 80424, Taiwan; ^4^Department of Occupational Therapy, I-Shou University, No. 8, Yida Road, Jiaosu Village, Yanchao District, Kaohsiung 82445, Taiwan; ^5^Department of Physical Therapy and Rehabilitation, Rehabilitation Assistive Device Center, Da-Chien General Hospital, No. 6, Shin Guang Street, Miaoli City 36049, Taiwan

## Abstract

*Objectives*. To conduct a systematic review comparing the effects of Kinesio taping with McConnell taping as a method of conservative management of patients with patellofemoral pain syndrome (PFPS). *Methods*. MEDLINE, PUBMED, EMBASE, AMED, and the Cochrane Central Register of Control Trials electronic databases were searched through July 2014. Controlled studies evaluating the effects of Kinesio or McConnell taping in PFPS patients were retrieved. *Results*. Ninety-one articles were selected from the articles that were retrieved from the databases, and 11 articles were included in the analysis. The methods, evaluations, and results of the articles were collected, and the outcomes of patellar tapings were analyzed. Kinesio taping can reduce pain and increase the muscular flexibility of PFPS patients, and McConnell taping also had effect in pain relief and patellar alignment. Meta-analysis showed small effect in pain reduction and motor function improvement and moderate effect in muscle activity change among PFPS patients using Kinesio taping. *Conclusions*. Kinesio taping technique used for muscles can relieve pain but cannot change patellar alignment, unlike McConnell taping. Both patellar tapings are used differently for PFPS patients and substantially improve muscle activity, motor function, and quality of life.

## 1. Introduction

Patellofemoral pain syndrome (PFPS) is one of the most common knee problems, predominantly in women [1]. The incidence rate of PFPS among athletes is 25%, which is higher than that of the general population [2]. PFPS is caused by repetitive stress on the musculotendinous structures which surround the knee and is aggravated in athletes by cycling and running [3]. The symptoms often occur in athletes because of increased intra-articular stress on the patellofemoral joint and are often caused by abnormal biomechanics of athletes, particularly during drop landing with the knee valgus [1]. PFPS is characterized by diffuse pain in the front of the knee that typically occurs when the individual ascends and descends stairs, squats, or sits for prolonged periods of time [3]. PFPS patient suffers excessive stress on patellofemoral joint in cases of abnormal structures of the lower extremities, such as deteriorated hip rotation control, increased feet pronation, femoral anteversion, and tibial rotation [4, 5]. Vastus medialis oblique (VMO) weakness and VMO and iliotibial band tightness through anatomical correlations to the patella cause lateral force vector and patellofemoral joint stress [6, 7]. An imbalance in muscle activities of VMO and vastus lateralis (VL) muscles leads to lateral patellar tracking during knee extension [8]. Thus, the multifactorial etiology of PFPS is associated with the musculotendinous structures of the knee.

Various conservative treatments, including therapeutic exercise, muscle strengthening exercises, muscle stretching, electrotherapy, knee bracing, and patellar taping, exist to treat PFPS [1]. Clinically, the purpose of the treatments is to achieve pain relief by correcting the patellar alignment so that it moves in the trochlear groove. The treatments often include stretching of lateral tight muscle (tensor fascia latae and iliotibial band) and strengthening of weak muscles (VMO muscle) around the patella to promote active medial stability and involve patella wearing during patella wearing [3]. Previous studies show promising results in alleviating the symptoms of PFPS, but applicable conservative treatments require further investigation [3, 5, 9]. Athletes, especially female athletes, often suffer from PFPS during training or racing. Gender-related anatomical structures of lower extremity, such as large *Q* angle of knee and quadriceps muscle weakness, cause high incidence of PFPS [2, 4]. PFPS often influences their sports performance and training efficiency. Surgery is another option for PFPS patients, and lateral tissue release and arthroscopy are used to treat patellar malalignment [1]. The time required to heal the affected knee postoperatively can influence an athlete's decision to return to sports [2]. However, some studies that compared the surgery with conservative treatments found no additional effect to functional recovery with surgery [1, 5].

Athletic taping is a conservative treatment approach for PFPS athletes, with the McConnell taping technique and the Kinesio taping method being the most popular tapings. The McConnell taping, first presented by Jenny McConnell in 1984, has been used to correct the abnormal patella position in athletes with PFPS [9, 10]. It gained popular acceptance for the prevention of sport injuries in a race. Athletes with PFPS were instructed to apply the patellar taping on the knee while engaged in sports activities and to wear the tape after the training or competition [9, 10]. McConnell taping was applied to correct patellar medial glide or tilt and revealed positive effects in pain for PFPS relief among previous studies [1, 9, 10]. Kinesio taping, introduced by Kenzo Kase in 1990, has recently become increasingly popular among athletes. Because of its flexibility, it can be applied to manage VMO and VL muscle imbalance [10]. Kinesio taping and McConnell taping have different principles and methods when used to treat PFPS, and no studies have explored the differences between the two methods. Therefore, this study used a systematic review and meta-analysis to compare the effects of Kinesio and McConnell patellar tapings in patients with PFPS.

## 2. Methods

### 2.1. Study Selection

The keywords* Kinesio taping*,* McConnell taping*,* taping*,* patellofemoral pain*, and* knee pain* were used as queries in MEDLINE, PUBMED, EMBASE, AMED, and the Cochrane Central Register of Control Trials electronic databases through January 2014. Non-English articles, case reports, and review studies were excluded. The inclusion criteria were articles in which a controlled study was described, patients were diagnosed with PFPS, and an experimental group received an intervention of Kinesio taping or McConnell taping. Two reviewers with more than 10 years of experience on sports medicine screened the articles to identify those that met the study criteria. The methods, evaluations, and results of the articles were collected, and the outcomes of Kinesio taping and McConnell taping were analyzed. Finally, the Jadad quality score was used to grade the quality of the articles by the reviewers [11]. The Jadad scale contains five items that are graded on a 5-point scale to assess the methodological quality of the article. A higher grade indicates that an article has higher quality.

### 2.2. Data Collection and Meta-Analysis

The extracted data of recruited articles were recorded by two reviewers, and statistical analysis was performed with the MedCalc software (MedCalc, Mariakerke, Belgium) for meta-analysis. The means and standard deviations of continuous outcome in the articles were analyzed to estimate the standardized mean difference and 95% confidence intervals (CI). Rosenthal's file drawer method was used to determine publication bias, which influences the result of meta-analysis when the fail-safe number is greater than the tolerance level. Positive standardized mean difference is in favor of patellar taping. A total effect was calculated by a total random effect model to assume the outcome effects of Kinesio taping and McConnell taping. Although no significant heterogeneity occurred, a total fixed effect model was used. Homogeneity was measured with the Cochran *Q* test and was statistically significant when *P* > 0.05 or *I* ^2^ < 50%. The file drawer analysis was used to explore publication bias. The effects of various assessment outcomes and the total effects were used in subgroup analysis to examine the effects of the patellar tapings. The grading of effect size was based on Cohen's rule and expressed as small (standardized  mean  difference = 0.2–0.5), moderate (standardized  mean  difference = 0.5–0.8), and large (standardized mean difference > 0.8) effect.

## 3. Results

### 3.1. Study Characteristics

**Figure 1:**
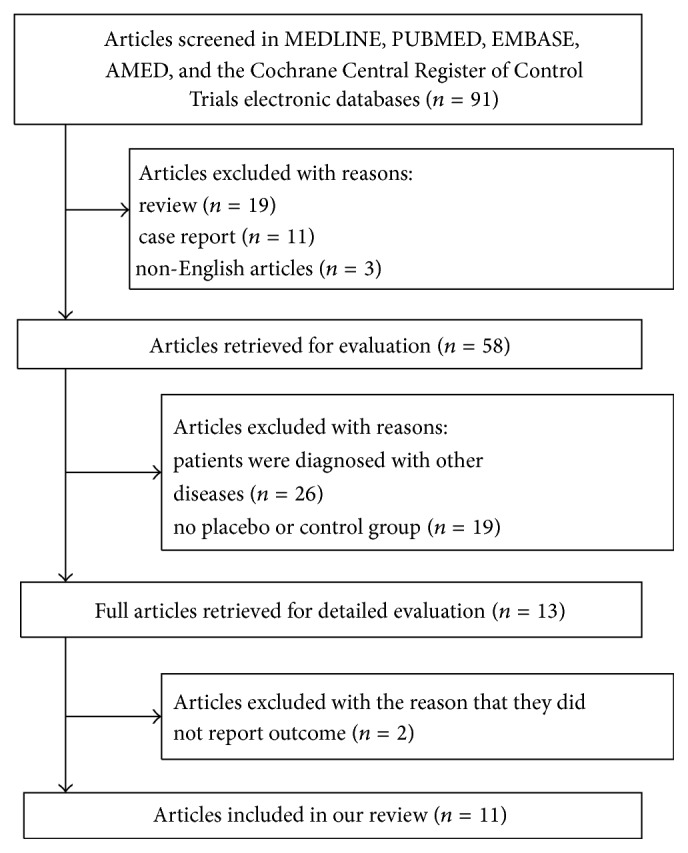
Flowchart of article search.

Ninety-one articles were retrieved from electronic databases. After the experts reviewed the abstracts and excluded articles, 11 articles were included in this study (Figure 1) [12–22]. Jadad quality scores must be 3–5 as shown in Table 1, and all studies were classified as high quality. The study of Campolo et al. [12] included two experimental groups, based on Kinesio taping and McConnell taping. Five studies used Kinesio Tex Tape (Kinesio Holding Corporation) to treat PFPS [12, 14–16, 18]. Seven studies used McConnell taping to treat PFPS. Fixing tape was used to prevent skin from slipping, and athletic tape, such as Endura-Fix tape (Endura, Inc.) [13], Protape (Norgesplaster, Inc.) [19], Leukotape P rigid tape (BSN-JOBST, Inc.) [21], and Hypafix tape (Smith & Nephew DonJoy, Inc.) [17, 22], was attached to the patella. Three studies used an intervention that combined patellar taping with neuromuscular retraining exercises. The exercises included stretching the iliotibial band and the tensor fasciae latae, isometric contraction exercises of the hamstring and quadriceps, isotonic contraction exercises of the hip adductors, hip, and gluteus medius, and maximus, open chain exercises (e.g., straight leg raises and terminal knee extension), and closed chain exercises (minisquats and single-leg stance) [14, 15, 19].

### 3.2. McConnell and Kinesio Tapings

**Figure 2:**
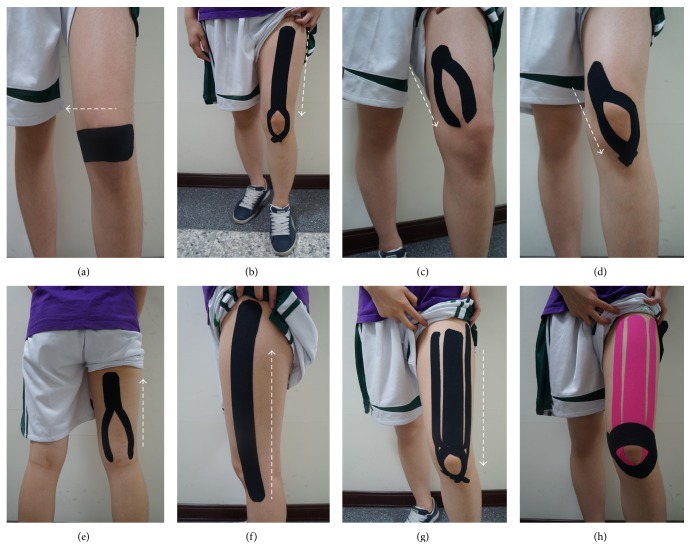
The applications of Kinesio taping for PFPS patients: white arrow is the sticking aspect, and available tension of tape is used over affected muscle, finishing with no tension. (a) I-shaped Kinesio tape with 50–75% tension is applied as McConnell taping, and the tape is covered with patellar to pull medially. Y-shaped tape with 10–15% tension is applied on quadriceps (b) and VMO muscle (c, d) to improve proprioceptive stimulation and muscle strength. The inverse methods of hamstring muscle covering Y-shaped tape (e) and iliotibial band covering I-shaped tape (f) with 10–15% tension could relieve the muscle tightness. Three Y-shaped tapes are applied to the quadriceps muscle to increase the facilitated tension (g). After finishing muscular tapping, 2 I-shaped tapes are applied around patella for patellar fixation (h).

In McConnell taping, the athletic tape is used to correct the patellar alignment and anchored over the patella to end at the medial knee [21]. The patella is manually moved medially and maintained in medial tilt with the athletic tape [23]. In contrast, Kinesio taping is a multiform approach (Figure 2). Kinesio tape is applied to correct the patellar position, to improve proprioceptive stimulation for VMO muscle weakness, and to relieve muscle tension from tightness of the VL muscles, hamstring muscles, and the iliotibial band [15]. Lan et al. [18] imitated the medial glide and medial tilt technique of McConnell taping and applied Kinesio tape with enough force (50–75% tension) to medially shift the patella for mechanical correction (Figure 2(a)). The Y-shaped Kinesio tape was used for VMO and quadriceps muscle and applied with 10–15% tension (paper off tension) over the muscle origin toward the patella (Figures 2(b), 2(c), and 2(d)) [12, 14–16]. The iliotibial band was covered with an I-shaped tape (Figure 2(f)), and hamstring muscles were covered with a Y-shaped tape (Figure 2(e)). With the use of 10–15% tension of the Kinesio tape to stick from the insertion to the origin of the muscle was applied on the hamstring muscle and iliotibial band [14, 15].

### 3.3. Data Synthesis

#### 3.3.1. Assessments for Outcomes of Patellar Tapings

The outcomes of the 11 articles were recorded. The effects of treatment for the PFPS patients studied were estimated by using the following 7 categories.

*(1) Pain.* The visual analog scale (VAS) was used to assess pain before and after patellar taping [12–21] and to assess pain induced by actions such as prolonged sitting, kneeling, walking, squatting, and ascending and descending stairs or hill [15, 16]. 

*(2) Muscle Flexibility*. The flexibility of the knee muscles was assessed to measure the muscular tightness of patients with PFPS. A goniometer was used to measure the range of motion to assess the degree of hamstring tightness. The patients lay supine on the bed, with the hips fixed to avoid rotation and the knees maintained at 90-degree angles. The evaluator kept the axis of the goniometer on the femoral condyle and aligned the mobile arms to the midline of the thigh and calf. The range of motion in the knee in a passive state was recorded [15]. Ober's test was used to assess the degree of tightness in the iliotibial band and in the tensor fasciae latae. Patients lay on their sides with their backs facing the evaluator. The hip was passively abducted and extended and subsequently adducted under pressure. The distance between the patella and the bed was measured [15]. 

*(3) Patellar Alignment*. Patellar alignment was assessed in the patellar position in the trochlear groove. A modified Vernier caliper was used to test the position of the patella. The patient lay on the bed, with the knee bent at a 20-degree angle. The modified Vernier caliper was used to orient the medial and lateral femoral condyle as well as the inner and outer edges of the patella. The 4 points were recorded to capture the patellar alignment [15]. Derasari et al. [17] used a 1.5 Tesla magnetic resonance imager (CV-9.1M4 or LX-9.1M4) to capture the image of the patellar position. The patients followed the rhythm of a metronome (35 bits/min) to repeat knee extension and flexion. The images were processed to calculate the 3-axis velocity of displacement and rotation of the femur, tibia, and patella to present the patellar movements (e.g., medial and lateral shifting, inferior and superior shifting, and anterior and posterior shifting) [17]. Tangential radiography was also used to measure the lateral patellofemoral angle, lateral patellar displacement, and *Q* angle in the X-ray images [18]. Patellar alignment was performed by flexing the knee to a 30-degree angle. 

*(4) Proprioception*. Proprioception was measured to represent the condition of the position sense receptors of the knee in patients with PFPS. The Biodex system dynamometer (Biodex Corporation) was used to assess the joint position sense. Blindfolded patients wore shorts, feet were bare, and they maintained their knees at a 90-degree sitting position. A tibia pad was placed on the anterior ankle above 3 cm, and an air bag cladding the lower leg was filled with air to the pressure of 40 mm Hg. Repeated passive and active joint angles of 20 degrees and 60 degrees were recorded for analysis [22]. An isokinetic dynamometer (Cybex 770, Lumex) was used to assess joint position sense [16]. The blindfolded patients felt the targeted position at the midpoint of the knee range (45 degrees) when their knees were passively moved from a 0-degree knee extension to a 90-degree knee flexion, at a constant velocity of 5 degrees/s. 

*(5) Motor Function*. Motor function was measured by assessing the exercise performances of patients with PFPS. A star excursion balance test (SEBT) was used to collect sagittal kinetic data on knee motion. The patients stood on a horizontal line before SEBT. One bare foot was affixed to the ground, and the other bare foot was moved as far forward as possible to complete the indicated direction. The reaching distances were recorded [21]. A Kinesthetic Ability Trainer (KAT 3000, Berg) was used to assess static and dynamic balance [16]. The patients who had their eyes open were asked to concentrate on the target on the screen and maintain balance for 30 s. The balance index scores for static and dynamic balance were then calculated. A triple jump test requiring three consecutive hops and a step test requiring a 25-cm ascent and descent were conducted to assess the function of the leg of a patient with PFPS [14]. A motion-analysis system (Reality Motion Systems, GmbH, Germany) was used to capture 3-dimensional coordinates of superficial reflective markers around the knee and to analyze the knee extensor moment and patellofemoral joint reaction force during single-leg squatting [20]. The Kujala scale is a self-reported questionnaire, which ranges from 0 to 100. It is also used to analyze the motor function of the knees [14, 15]. 

*(6) Muscle Activity*. Muscle activity was assessed by measuring changes of muscle strength and the electromyographic signal of knee extensor muscle. A manual muscle test is a common test to assess knee extension strength and is based on a 5-point scale [14]. Electromyography was used to measure muscle activity. Onset activity of the electromyography signal was recorded to determine the latency time of a recruiting motor unit to perform muscle activity [19]. Other assessment data were the percentage of the maximal voluntary isometric contraction of VMO and VL, which were assessed at a 60-degree knee joint flexion during squatting, and the ratio of VMO and VL was calculated [13]. Cybex was also used to determine 60 degrees/s and 180 degrees/s angular velocity for assessing the isokinetic strength of the knee extensors [16, 19]. Cybex can measure muscle strength to determine mean torque. 

*(7) Quality of Life*. The Medical Outcomes Study Short Form 36 (SF-36) questionnaire, in addition to scoring knee pain, knee function, and self-emotion, was used to assess the affection of physical and mental health status of the PFPS patients [14].

#### 3.3.2. Outcomes of the Patellar Tapings

The study results of Campolo et al. [12], Akbaş et al. [15], and Lan et al. [18] indicated that applying Kinesio taping to PFPS patients significantly reduces pain and increases muscle flexibility when compared with the control groups. In addition, Lan et al. [18] determined that they imitated McConnell taping to approach patellar alignment correction by using Kinesio tape, but no statistically significant differences between the experimental and control groups were observed. Kaya et al. [19] and Mostamand et al. [20] indicated that applying McConnell taping to PFPS patients significantly reduced pain, compared with the control groups. Compared with control groups, the study results of Lee and Cho [13] revealed that the muscle activity of VMO statistically significantly increased, but no significant difference was observed by Kaya et al. [19]. Derasari et al. [17] indicated that the application of McConnell taping to PFPS patients showed a more significant increase in the patellar inferior shifting than that in the control group. However, no statistically significant differences in proprioception and motor function were observed between the experimental and control groups [21, 22].

**Figure 3:**
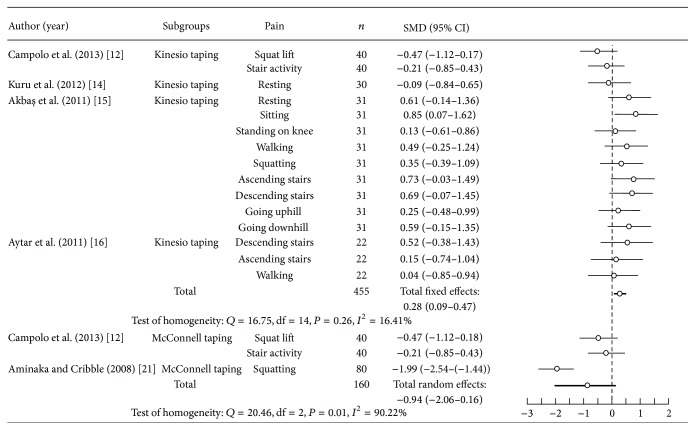
Pooled estimate of effects in pain with Kinesio taping and McConnell taping.

The fail-safe number (value = 129) was higher than the tolerance level (value = 65), and the publication bias did not influence the results of meta-analysis. Pain assessment before and after patellar taping was reported in five articles [12, 14–16, 21]. A significantly heterogeneous pain change was found for patellar taping in PFPS (*n* = 615; *P* < 0.05; *Q* = 81.42; *I* ^2^ = 79.12%; standardized mean difference = 0.08; 95% CI, −0.28–0.44). There was significant homogeneity in pain reduction with Kinesio taping (*P* > 0.05; *Q* = 16.75; *I* ^2^ = 16.41%) and significant heterogeneity in pain reduction with McConnell taping (*P* < 0.05; *Q* = 20.46; *I* ^2^ = 90.22%) in the subgroup analysis (Figure 3). Among PFPS patients that used Kinesio taping, there was a significant total effect in pain (*n* = 455; standardized mean difference = 0.28; 95% CI, 0.09–0.47; *I* ^2^ = 16.41%), which was superior to McConnell taping (*n* = 160; standardized mean difference = −0.94; 95% CI, −2.06–0.16; *I* ^2^ = 90.22%). Therefore, the result of meta-analysis showed that the use of Kinesio taping for PFPS patients had a small effect in pain relief.

**Figure 4:**
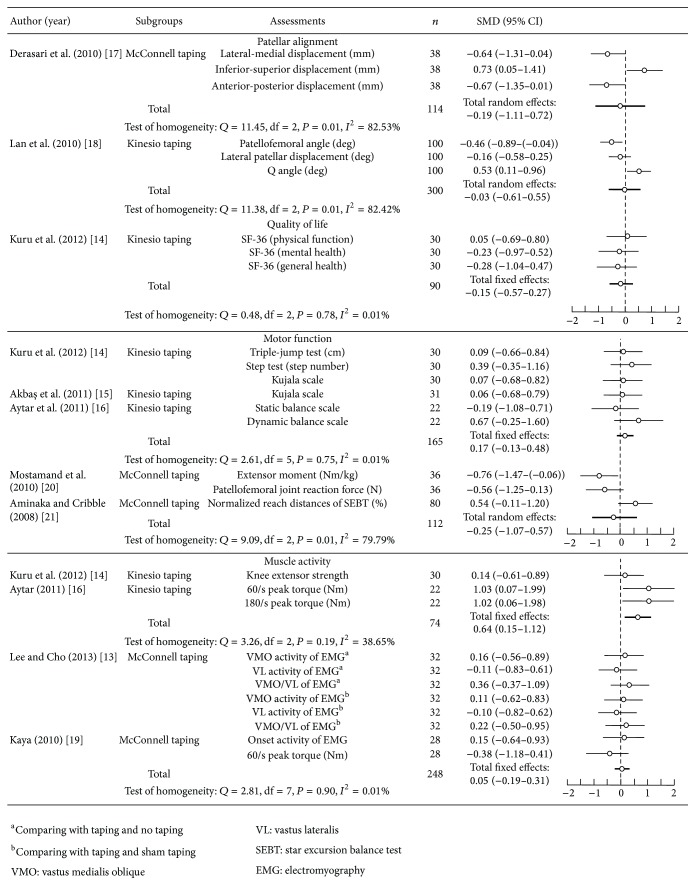
Pooled estimate of effects in outcome measures.

Figure 4 shows the results of two articles reporting the results of patellar alignment [17, 18], five articles reporting the outcomes of motor function [14–16, 20, 21], and four articles reporting the outcomes of muscle activity [13, 14, 16, 19]. The study findings were significantly heterogeneous in patellar alignment (*n* = 414; *P* < 0.05; *Q* = 23.32; *I* ^2^ = 78.56%; standardized mean difference = −0.11; 95% CI, −0.56–0.35) and significantly homogeneous in motor function (*n* = 277; *P* > 0.05; *Q* = 14.19; *I* ^2^ = 43.62%; standardized mean difference = 0.02; 95% CI, −0.22–0.27) and in motor function (*n* = 322; *P* > 0.05; *Q* = 1.99; *I* ^2^ = 0.01%; standardized mean difference = 0.09; 95% CI, −0.13–0.31) for patellar taping on PFPS. In the subgroup analysis, the results in patellar alignment (*P* < 0.05; *Q* = 11.38; *I* ^2^ = 82.53%) of using Kinesio taping and that in patellar alignment (*P* < 0.05; *Q* = 11.45; *I* ^2^ = 82.42%) and motor function (*P* < 0.05; *Q* = 9.09; *I* ^2^ = 79.79%) of using McConnell taping had significantly heterogeneous results. The results in motor function (*P* > 0.05; *Q* = 2.61; *I* ^2^ = 0.01%) and muscle activity (*P* > 0.05; *Q* = 3.26; *I* ^2^ = 38.65%) of using Kinesio taping and that in muscle activity (*P* > 0.05; *Q* = 2.81; *I* ^2^ = 0.01%) of using McConnell taping had significant homogeneity. The total effects in muscle function (*n* = 165; standardized mean difference = 0.17; 95% CI, −0.13–0.48; *I* ^2^ = 0.01%) and muscle activity (*n* = 74; standardized mean difference = 0.64; 95% CI, 0.15–1.12; *I* ^2^ = 38.65%) of patients with PFPS who used Kinesio taping had significantly more improvements (motor function: *n* = 112; standardized mean difference = −0.25; 95% CI, −1.07–0.57; *I* ^2^ = 79.79%; muscle activity: *n* = 248; standardized mean difference = 0.05; 95% CI, −0.19–0.31; *I* ^2^ = 0.01%) than the PFPS patients who used McConnell taping. The results of meta-analysis demonstrated that there were small increases in motor function improvement and moderate increases in muscle activity among PFPS patients who used Kinesio taping.

## 4. Discussion

Kinesio taping has favorable properties and is composed of waterproof and ventilative material [16]. Patients like Kinesio taping because its favorable adhesive properties facilitate easy use and prevent allergic reactions; thus, Kinesio taping is in widespread clinical use [24]. Kinesio taping involves affixing Kinesio tape to the skin folds to increase the space between the muscle and fascia [15, 16]. Kinesio tape can increase local blood or lymphatic circulation [12, 14]. There are two approaches to Kinesio taping. First, the tape can be applied in the direction of muscle contraction from muscle origin to the insertion; this method facilitates the contraction of injured muscles [16, 24]. The second approach involves adhering the tape in the opposite direction of muscle contraction from the muscle insertion to origin. It prevents muscle overuse to provide excessive muscle tension [24, 25]. McConnell taping uses a different approach and material for affixing the rigid tape to correct patellar alignment. Three components of patellar orientation, such as glide component, tilt component, and rotation component, were assessed before the patellar tape. The principle of the taping is to restrict abnormal patella tracking, thus reducing joint friction and oppression of the injured tissue [12, 20]. However, Kinesio taping uses elastic tape to provide active muscle contraction and increase the pull tension of the patella. Therefore, the materials, uses, and principles of the two patellar taping approaches differ [10]. Lim et al. [26] indicated that the elastic material of Kinesio tape is similar to the skin and soft tissue and its elasticity allows it to elongate to 130% to 140% of its initial length. When Kinesio tape is applied with different methods on muscle, Kinesio taping provides tension force to pull or inhibit muscle contraction. The skin folds caused by tape elasticity can increase local lymph and blood circulation, and metabolic substances can be removed [26]. These factors may be possible causes of the analgesic effect.

Kinesio tape consists of thin, cotton, and acrylic-acid-containing porous fabric. The tape is a unique and nonlatex adhesive patch [27]. Akbaş et al. [15] applied Kinesio taping to PFPS patients by adhering Kinesio tape onto the VMO muscle to increase proprioception input and to facilitate muscle contraction. In addition, they applied the tape over the iliotibial band, tensor fasciae latae, and VL muscle to inhibit muscle tension and relax the muscle. Lan et al. [18] used Kinesio taping to imitate the use of McConnell taping. They used the hands to push the patella to the inside and applied Kinesio tape to fix the patella. Subsequently, PFPS patients repeatedly stepped up and down on an 8-inch step, and induced pain was assessed. The results revealed that Kinesio taping reduces PFPS-associated pain but does not change patellar alignment. González-Iglesias et al. [28] believe that Kinesio taping can be applied at 50% to 85% tensions on the skin to restrict partial or full joint motion, but the taping tension was insufficient to correct the patellar alignment. Kinesio taping pulls the skin to increase the gap between the skin and muscle, reduces tissue edema, and promotes blood and lymphatic circulation [15, 24, 29]; thus, Kinesio taping effectively relieves pain. Positive effects of the taping were also seen on reduced swelling and muscle spasms of PFPS patients [15, 24, 25]. Several studies suggest that Kinesio taping is useful in treating acute sports injuries because it immediately reduces pain, improves muscle contracture [24, 27], and accelerates athletes' return to normal activity [16]. However, studies proving the effects of Kinesio taping are still rare, and their results are inconsistent [27, 30].

In McConnell taping, under tape that exerts no tension on the skin of the patient is applied, followed by a rigid tape to correct the patellar position [12, 19–22]. Under tape is a flexible and scalable patch composed of nonwoven polyester fabric material [19–22]. It can be used in most circumstances, including when athletes are sweating or bathing and other moist situations [12, 19, 20]. McConnell taping involves the use of several types of rigid tape that have distinct characteristics. Hypafix tape and Endura-Fix tape are malleable and water-resistant patches [13, 17, 22]. Because the fabric patch is glued by using an adhesive, Hypafix tape is suitable for use on the skin [17, 22]. Leukotape P rigid tape is a strong adhesive woven rayon cloth patch that is composed of zinc oxide and features high elasticity [21]. Derasari et al. [17] applied McConnell taping patients by covering the quadriceps of PFPS patients with Hypafix tape in a relaxed supine position. The medial edge of the patella was pushed outward and fixed with the tape. The patients walked for 5 minutes to feel comfortable and adapt to the McConnell taping before the experiment. The strongest correlation between the change in lateral-medial displacement of patella before and after the taping was found [17]. Callaghan et al. [22] asked PFPS patients to relax their knees in a supine posture. They used Hypafix tape to determine the patellar alignment, and two tape layers covered the knee. The tension-free tape of the lower layer was stuck on to the patella, and the average tensioned tape of the upper layer covered the patella to ensure that it remained in the middle of knee. The use of McConnell taping in the study of Aminaka and Gribble [21] involved affixing 15 cm pieces of Cover-Roll tape to the knees of PFPS patients. A 12 cm piece of Leukotape P rigid tape was applied on the inward patella and fixed to its medial edge to provide medial force from the femoral condyle and to push the patella to maintain it in the femoral groove. These study results also indicate that McConnell taping corrects patellar alignment and tracking [17] but does not improve the motor function and proprioception of PFPS patients [21, 22]. Callaghan et al. [22] collected and analyzed assessment results on PFPS patients with poor proprioception and observed that McConnell taping improves proprioception. The results indicate that various materials and the identical approach of McConnell taping are effective in treating particular problems of PFPS patients, but the correlation between correct patellar alignment and functional outcomes need further study.

The results of several studies indicate that Kinesio and McConnell taping can reduce pain in PFPS patients [12, 14–16, 18–21]. The mechanism of Kinesio taping that affects pain in patients with PFPS remains unclear [18], and evidence of the underlying mechanism by which McConnell taping affects patients with PFPS is also weak [31]. Most studies consistently indicate that Kinesio taping or McConnell taping can stimulate cutaneous mechanoreceptors and improve knee proprioception [12, 16]. The sensory input can increase feedback to the central nervous system and cause pain to decrease. Thus, taping seems to involve the gate control theory as a cause of pain modulation [12, 15, 21]. The results of Kuru et al. [14] and Akbaş et al. [15] indicated significant pain relief in PFPS patients after Kinesio taping. They inferred that Kinesio taping can facilitate quadriceps muscle contraction and that the increased muscle strength can provide dynamic patellar stability to maintain normal patellar tracking, thus reducing pain. But some results indicate a nonsignificant difference in muscle activity, including muscle strength and electromyography findings, between Kinesio taping groups and control groups [14, 16]. Although Kinesio taping is applied to the affected muscle, and the approach can provide excessive muscle tension, the result of increased muscle activity may not be the reason for pain relief. Kinesio taping might increase sensory input in the skin and activate proprioception to increase muscle force control [32]. The result reported by Aytar et al. indicated a nonsignificant change in knee joint proprioception of PFPS patients, compared with a control group [16]. Chang et al. [33] reported that Kinesio taping of the muscles can help joint mechanoreceptors improve joint position sense and might help cutaneous mechanoreceptors stimulate the muscle spindle or Golgi tendon organ. Kinesio taping is effective in controlling patellar tracking through increased muscle force sense, and that might the mechanism of pain relief in PFPS patients. McConnell taping to correct patellar alignment also cause pain relief in PFPS patients [19–21]. Kaya et al. [19] and Mostamand et al. [20] indicate that quadriceps muscle contraction, particularly the VMO muscle, can be improved by using McConnell taping, and the type of patellar alignment can be corrected to enable pain-free exercise. The taping affected pain-free strengthening of knee extensors during neuromuscular retraining exercises [19]. One limitation of McConnell taping is that PFPS patients must apply the tape before exercise and remove it after exercise [19]. Lan et al. [18] reported that the type of patellar taping should be considered on an individual basis for clinical use; in other words, the specific problems of each patient must be considered. Compared with McConnell taping, the use of Kinesio taping is more accepted in PFPS patients. Thus, the use of Kinesio taping for athletes has become widespread in recent years.

Many studies indicate that therapeutic exercises for PFPS patients emphasize VMO strengthening and patellar medial stabilization [14, 34]. Open and closed kinetic chain exercises appeared to be efficacious methods of increasing VMO muscle strength and improving quadriceps muscle imbalances [15, 35, 36]. Combining strengthening exercises and patellar taping had significant effects in pain relief and improvement of muscle activity and function [14, 15, 19]. Results from Kuru et al. [14] and Akbaş et al. [15] demonstrated that Kinesio taping combined with strengthening exercises can improve training effects of the VMO muscle [14, 15]. They concluded that the use of Kinesio taping on VMO muscle could activate cutaneous mechanoreceptors to facilitate muscle contraction. Kinesio taping combined with strengthening exercise can enhance the awareness of the correct muscle contraction during exercise training in PFPS patients [37]. Akbaş et al. also indicated that Kinesio taping can increase the subcutaneous space to improve blood flow, while affixing Kinesio tape to VMO muscle [15]. They inferred that improved blood circulation is another possible effect on muscle function. Kaya et al. [19] used McConnell taping combined with strengthening exercise to treat PFPS and also found positive effects in pain reduction and muscle activity. They indicated that McConnell taping corrected patellar alignment in femoral groove and reduced pain during exercise of VMO muscle contraction. However, the McConnell taping needed to be removed for the skin to recover after the strengthening exercise. Prolonged McConnell taping has the side effects of skin discomfort and allergic reaction, whereas Kinesio taping can be continuously used for 3 to 7 days, and keep the therapeutic effects for daily usage [14]. Although prolonged Kinesio taping also caused allergic side effects, its convenience is recognized among PFPS patients.

## 5. Conclusion

Kinesio taping and McConnell taping are two types of patellar taping used to treat patients with PFPS. Kinesio taping can be applied to muscles to relieve pain, but there is a lack of evidence on effect of the effects of taping on patellar alignment correction. McConnell taping can adjust patellar alignment and tracking but does not improve proprioception and motor function for PFPS. Based on the current review study, both types of taping significantly improve muscle activity, motor function, and quality of life, benefits which are possibly facilitated by pain relief. However, further research examining the mechanism of pain relief is required.

## Figures and Tables

**Table 1:** Characteristics of 11 articles.

Author (year)	*n*	Age	Group (samples)	Intervention	Treatment duration	Assessments	Assessment time	Results	Jadad scale
Campolo et al. (2013) [12]	20	24.0	Experimental group A (*n* = 20) Experimental group B (*n* = 20) Control group (*n* = 20)	Kinesio taping McConnell taping No taping	Immediate taping	VAS	Before and after taping	Pain decreased in Kinesio taping^*∗*^ and McConnell taping	3

Lee and Cho (2013) [13]	16	31.6	Experimental group (*n* = 16) Control group A (*n* = 16) Control group B (*n* = 16)	McConnell taping Sham tape^a^ No taping	Immediate taping	EMG	Before and after taping	Muscle activity improved^*∗*^	4

Kuru et al. (2012) [14]	30	36.5	Experimental group (*n* = 15) Control group (*n* = 15)	Kinesio taping + exercise Electrostimulation + exercise	6-week taping	VAS, MMT, SF-36, triple jump test, step test, Kujala scale	Before and after taping	Pain decreased Muscle activity and motor function increased, and quality of life improved	3

Akbaş et al. (2011) [15]	31	44.9	Experimental group (*n* = 15) Control group (*n* = 16)	Kinesio taping + exercise No taping	6-week taping	VAS, hamstring tightness, Ober's test, modified Vernier caliper, Kujala scale	3 weeks and 6 weeks before taping	Pain decreased^*∗*^ Motor function increased^*∗*^ Patellar alignment and muscle flexibility improved	5

Aytar et al. (2011) [16]	22	24.1	Experimental group (*n* = 12) Control group (*n* = 10)	Kinesio taping Sham tape^a^	Immediate taping	VAS, muscle strength, joint position sense, static and dynamic balance, isokinetic strength	Before and after taping	Pain decreased Muscle activity, proprioception, and motor function improved	4

Derasari et al. (2010) [17]	38	28.7	Experimental group (*n* = 19) Control group (*n* = 19)	McConnell taping No taping	Immediate taping	MRI	Before and after taping	Patellar alignment improved^*∗*^	3

Lan et al. (2010) [18]	100	42.0	Experimental group (*n* = 66) Control group (*n* = 34)	Kinesio taping Sham tape^a^	Immediate taping	VAS, lateral patellofemoral angle, lateral patellar displacement, and *Q* angle	Before, intermediate, and after taping	Pain decreased^*∗*^ No change of patellar alignment	4

Kaya et al. (2010) [19]	28	24.2	Experimental group (*n* = 12) Control group^b^ (*n* = 16)	McConnell taping + exercise No taping	3-month taping	VAS, EMG, isokinetic strength	Before and after taping	Pain decreased^*∗*^ Muscle activity improved	3

Mostamand et al. (2010) [20]	36	27.1	Experimental group (*n* = 18) Control group^b^ (*n* = 18)	McConnell taping No taping	7-day taping	VAS, knee extensor moment, and patellofemoral joint reaction forces	Before and after taping	Pain decreased^*∗*^ Motor function improved	5

Aminaka and Gribble (2008) [21]	80	20.8	Experimental group (*n* = 40) Control group (*n* = 40)	McConnell taping No taping	Immediate taping	VAS, SEBT	Before and after taping	Pain decreased	3

Callaghan et al. (2008) [22]	64	31.9	Experimental group (*n* = 32) Control group (*n* = 32)	McConnell taping No taping	Immediate taping	Joint position sense	Before and after taping	No change of proprioception	3

^a^Nonresponsive or nonstretched taping, ^b^healthy subjects. ^*∗*^ *P* < 0.05, experimental group versus control group.

VAS, visual analog scale; EMG, electromyography; MMT, manual muscle test; MRI, magnetic resonance imaging; SEBT, star excursion balance test.

## References

[1] Powers C. M. (1998). Rehabilitation of patellofemoral joint disorders: a critical review. *The Journal of Orthopaedic and Sports Physical Therapy*.

[2] Witvrouw E., Lysens R., Bellemans J., Cambier D., Vanderstraeten G. (2000). Intrinsic risk factors for the development of anterior knee pain in an athletic population: a two-year prospective study. *The American Journal of Sports Medicine*.

[3] Wilson T., Carter N., Thomas G. (2003). A multicenter, single-masked study of medial, neutral, and lateral patellar taping in individuals with patellofemoral pain syndrome. *The Journal of Orthopaedic and Sports Physical Therapy*.

[4] Cibulka M. T., Threlkeld-Watkins J. (2005). Patellofemoral pain and asymmetrical hip rotation. *Physical Therapy*.

[5] Sutlive T. G., Mitchell S. D., Maxfield S. N. (2004). Identification of individuals with patellofemoral pain whose symptoms improved after a combined program of foot orthosis use and modified activity: a preliminary investigation. *Physical Therapy*.

[6] Wilson N. A., Press J. M., Koh J. L., Hendrix R. W., Zhang L.-Q. (2009). In vivo noninvasive evaluation of abnormal patellar tracking during squatting in patients with patellofemoral pain. *The Journal of Bone and Joint Surgery—American Volume*.

[7] Cowan S. M., Bennell K. L., Hodges P. W., Crossley K. M., McConnell J. (2001). Delayed onset of electromyographic activity of vastus medialis obliquus relative to vastus lateralis in subjects with patellofemoral pain syndrome. *Archives of Physical Medicine and Rehabilitation*.

[8] Pal S., Besier T. F., Draper C. E. (2012). Patellar tilt correlates with vastus lateralis: vastus medialis activation ratio in maltracking patellofemoral pain patients. *Journal of Orthopaedic Research*.

[9] Clifford A. M., Harrington E. (2013). The effect of patellar taping on squat depth and the perception of pain in people with anterior knee pain. *Journal of Human Kinetics*.

[10] Osorio J. A., Vairo G. L., Rozea G. D. (2013). The effects of two therapeutic patellofemoral taping techniques on strength, endurance, and pain responses. *Physical Therapy in Sport*.

[11] Clark H. D., Wells G. A., Huët C. (1999). Assessing the quality of randomized trials: reliability of the Jadad scale. *Controlled Clinical Trials*.

[12] Campolo M., Babu J., Dmochowska K., Scariah S., Varughese J. (2013). A comparison of two taping techniques (kinesio and mcconnell) and their effect on anterior knee pain during functional activities. *International Journal of Sports Physical Therapy*.

[13] Lee S. E., Cho S. H. (2013). The effect of McConnell taping on vastus medialis and lateralis activity during squatting in adults with patellofemoral pain syndrome. *Journal of Exercise Rehabilitation*.

[14] Kuru T., Yaliman A., Dereli E. E. (2012). Comparison of efficiency of Kinesio taping and electrical stimulation in patients with patellofemoral pain syndrome. *Acta Orthopaedica et Traumatologica Turcica*.

[15] Akbaş E., Atay A. Ö., Yüksel I. (2011). The effects of additional kinesio taping over exercise in the treatment of patellofemoral pain syndrome. *Acta Orthopaedica et Traumatologica Turcica*.

[16] Aytar A., Ozunlu N., Surenkok O., Baltaci G., Oztop P., Karatas M. (2011). Initial effects of kinesio taping in patients with patellofemoral pain syndrome: a randomized, double-blind study. *Isokinetics and Exercise Science*.

[17] Derasari A., Brindle T. J., Alter K. E., Sheehan F. T. (2010). McConnell taping shifts the patella inferiorly in patients with patellofemoral pain: a dynamic magnetic resonance imaging study. *Physical Therapy*.

[18] Lan T.-Y., Lin W.-P., Jiang C.-C., Chiang H. (2010). Immediate effect and predictors of effectiveness of taping for patellofemoral pain syndrome: a prospective cohort study. *The American Journal of Sports Medicine*.

[19] Kaya D., Callaghan M. J., Ozkan H. (2010). The effect of an exercise program in conjunction with short-period patellar taping on pain, electromyogram activity, and muscle strength in patellofemoral pain syndrome. *Sports Health*.

[20] Mostamand J., Bader D. L., Hudson Z. (2010). The effect of patellar taping on joint reaction forces during squatting in subjects with patellofemoral pain syndrome (PFPS). *Journal of Bodywork and Movement Therapies*.

[21] Aminaka N., Gribble P. A. (2008). Patellar taping, patellofemoral pain syndrome, lower extremity kinematics, and dynamic postural control. *Journal of Athletic Training*.

[22] Callaghan M. J., Selfe J., McHenry A., Oldham J. A. (2008). Effects of patellar taping on knee joint proprioception in patients with patellofemoral pain syndrome. *Manual Therapy*.

[23] Crossley K., Bennell K., Green S., Cowan S., McConnell J. (2002). Physical therapy for patellofemoral pain: a randomized, double-blinded, placebo-controlled trial. *The American Journal of Sports Medicine*.

[24] Tsai C.-T., Chang W.-D., Lee J.-P. (2010). Effects of short-term treatment with kinesiotaping for plantar fasciitis. *Journal of Musculoskeletal Pain*.

[25] Kim H., Lee B. (2013). The effects of kinesio tape on isokinetic muscular function of horse racing jockeys. *Journal of Physical Therapy Science*.

[26] Lim C., Park Y., Bae Y. (2013). The effect of the kinesio taping and spiral taping on menstrual pain and premenstrual syndrome. *Journal of Physical Therapy Science*.

[27] Morris D., Jones D., Ryan H., Ryan C. G. (2013). The clinical effects of Kinesio Tex taping: a systematic review. *Physiotherapy Theory and Practice*.

[28] González-Iglesias J., Fernández-de-Las-Peñas C., Cleland J., Huijbregts P., Del Rosario Gutiérrez-Vega M. (2009). Short-term effects of cervical kinesio taping on pain and cervical range of motion in patients with acute whiplash injury: a randomized clinical trial. *Journal of Orthopaedic and Sports Physical Therapy*.

[29] Fu T.-C., Wong A. M. K., Pei Y.-C., Wu K. P., Chou S.-W., Lin Y.-C. (2008). Effect of Kinesio taping on muscle strength in athletes-a pilot study. *Journal of Science and Medicine in Sport*.

[30] Firth B. L., Dingley P., Davies E. R., Lewis J. S., Alexander C. M. (2010). The effect of kinesiotape on function, pain, and motoneuronal excitability in healthy people and people with achilles tendinopathy. *Clinical Journal of Sport Medicine*.

[31] Ernst G. P., Kawaguchi J., Saliba E. (1999). Effect of patellar taping on knee kinetics of patients with patellofemoral pain syndrome. *The Journal of Orthopaedic and Sports Physical Therapy*.

[32] Chang H.-Y., Chou K.-Y., Lin J.-J., Lin C.-F., Wang C.-H. (2010). Immediate effect of forearm Kinesio taping on maximal grip strength and force sense in healthy collegiate athletes. *Physical Therapy in Sport*.

[33] Chang H.-Y., Wang C.-H., Chou K.-Y., Cheng S.-C. (2012). Could forearm kinesio taping improve strength, force sense, and pain in baseball pitchers with medial epicondylitis?. *Clinical Journal of Sport Medicine*.

[34] McConnell J. (2002). The physical therapist's approach to patellofemoral disorders. *Clinics in Sports Medicine*.

[35] Irish S. E., Millward A. J., Wride J., Haas B. M., Shum G. L. K. (2010). The effect of closed-kinetic chain exercises and open-kinetic chain exercise on the muscle activity of vastus medialis oblique and vastus lateralis. *Journal of Strength and Conditioning Research*.

[36] Chang W. D., Huang W. S., Lee C. L., Lin H. Y., Lai P. T. (2014). Effects of open and closed kinetic chains of sling exercise therapy on the muscle activity of the vastus medialis oblique and vastus lateralis. *Journal of Physical Therapy Science*.

[37] Chen C.-H., Huang T.-S., Chai H.-M., Jan M.-H., Lin J.-J. (2013). Two stretching treatments for the hamstrings: proprioceptive neuromuscular facilitation versus kinesio taping. *Journal of Sport Rehabilitation*.

